# Supplementary material for: Machine Learning Methods for Automatic Segmentation of Images of Field- and Glasshouse-Based Plants for High-Throughput Phenotyping
Source: Plants (Basel). 2023 May 19;12(10):2035. doi: 10.3390/plants12102035 (PMC10224253; doi:10.3390/plants12102035)
Supplement: Supplementary file 1 [file plants-12-02035-s001.zip › plants-2329973-supplementary.pdf]

plants

SUPPLEMENTARY DATA

Table S1. Training, validation, and testing data the classification models

Features	Total Pixels	Training Data (pixels)	Validation Data (pixels)	Testing Data (pixels)
All Features (AF)	24,000,000	14,000,000	4,800,000	4,800,000
Selected Features (SF)	12,000,000	7,200,000	2,400,000	2,400,000

Table S2. Training, validation, and testing data for the different machine learning models

Test Dataset	Model	Processing time (s)	
		AF	SF
10	MLP	5.150	2.450
	SVM	132.158	86.163
	RF	81.225	44.581
15	MLP	9.443	2.931
	SVM	187.055	105.434
	RF	156.355	58.145
30	MLP	17.220	6.052
	SVM	277.474	155.181
	RF	188.553	98.336
